# Recommending Suitable Smart Technology Applications to Support Mobile Healthcare after the COVID-19 Pandemic Using a Fuzzy Approach

**DOI:** 10.3390/healthcare9111461

**Published:** 2021-10-28

**Authors:** Toly Chen, Yu-Cheng Wang

**Affiliations:** 1 Department of Industrial Engineering and Management, National Yang Ming Chiao Tung University, University Road, Hsinchu 1001, Taiwan; tolychen@ms37.hinet.net; 2 Department of Aeronautical Engineering, Chaoyang University of Technology, Taichung 41349, Taiwan

**Keywords:** smart technology, mobile healthcare, COVID-19 pandemic, calibrated fuzzy geometric mean, FTOPSIS

## Abstract

The COVID-19 pandemic seems to be entering its final stage. However, to restore normal life, the applications of smart technologies are still necessary. Therefore, this research is dedicated to exploring the applications of smart technologies that can support mobile healthcare after the COVID-19 pandemic. To this end, this study compares smart technology applications to support mobile healthcare within the COVID-19 pandemic with those before the pandemic, so as to estimate possible developments in this field. In addition, to quantitatively assess and compare smart technology applications that may support mobile healthcare after the COVID-19 pandemic, the calibrated fuzzy geometric mean (CFGM)-fuzzy technique for order preference by similarity to ideal solution (FTOPSIS) approach is applied. The proposed methodology has been applied to evaluate and compare nine potential smart technology applications for supporting mobile healthcare after the COVID-19 pandemic. According to the experimental results, “vaccine passport and related applications” and “smart watches” were the most suitable smart technology applications for supporting mobile healthcare after the COVID-19 pandemic.

## 1. Introduction

Smart technologies are applications of electronic devices or systems that can be connected to the Internet, used interactively, and are to some extent intelligent [1,2,3]. Smart technologies have been widely applied to mobile healthcare before the outbreak of the COVID-19 pandemic [4,5,6,7]. After the outbreak of the COVID-19 pandemic, a number of new smart technology applications have emerged. For example,

- Robots (or drones) help to build a communication with or send medicine to, a quarantined patient to reduce the burden on medical professionals and contain the contagion [8]. Robots (or drones) are also used to patrol in public spaces, observe and broadcast information to crowds, and monitor traffic more efficiently [9].
- In factories, voice commands or gestures are used to interact with machines to avoid spreading COVID-19 by touching the machines [10,11]. The same purpose can be achieved by using a smartphone to remotely control a machine [12].
- Workers wear smart wristbands or watches to detect their body temperature [13].
- In hotels, autonomous robots emit concentrated UV-C ultraviolet light to disinfect room keys, guest rooms, lobbies, gyms, and other public areas [14].
- In museums, wearable sensors are used to measure the proximity of visitors to ensure physical distance [15].
- App-based location-based services disperse users to avoid gatherings [16].

Obviously, smart technology applications to support mobile healthcare during the COVID-19 are different from those used before the pandemic. Some smart technologies (such as robots) are more commonly used, while others (such as apps that recommend restaurants with healthy diets) have lost public attention. With the increasing popularity of vaccination, many countries or regions are gradually unblocking. The COVID-19 pandemic seems to be entering its final stage. However, the impact caused by the COVID-19 pandemic will not disappear in a short time [17]. For the restoration of normal life, the applications of smart technologies are still necessary [18]. Therefore, this study intends to explore what kinds of smart technologies will be commonly applied to support mobile healthcare in the later stages of the pandemic.

The importance and contribution of this study is explained as follows. Suitable smart technology applications to mobile healthcare will change after the COVID-19 pandemic. In theory, some recent studies have found that the acceptance of smart technologies, such as smart robots, electronic medical records (evolved into vaccine passports), smart bracelets, and sociometric badges has become higher during the COVID-19 pandemic [19,20,21,22,23,24,25,26,27]. In contrast, some smart technologies, such as wireless medical sensor networks have been found to be impractical due to the inability to process a large amount of COVID-19 patient data [26]. In other words, after the COVID-19 pandemic, suitable smart technology applications to mobile healthcare will be different from those before the pandemic. This topic has not been thoroughly discussed in the literature. The present study aims to fill this gap. Practically speaking, many mobile healthcare providers are optimistic that the market size of smart technology applications will grow substantially, especially at the front end (client side) of mobile healthcare [28]. However, without distinguishing the changes in the acceptances of different smart technologies during the pandemic, their investments will be blind and not necessarily rewarding [29]. To these mobile healthcare providers, the findings of this study will be of reference value.

The procedure for this study is as follows. First of all, this study analyzes smart technology applications to support mobile healthcare within the COVID-19 pandemic and compares with those before the pandemic. Based on the comparison results, possible developments in the applications of smart technologies to support mobile healthcare in the later stages of the COVID-19 pandemic are estimated. However, the above discussion is qualitative and imprecise. A quantitative analysis is still necessary. To this end, from related literature and reports, some possible smart technology applications are listed and compared using a fuzzy multi-objective decision-making (MCDM) approach—the calibrated fuzzy geometric mean (CFGM) and fuzzy technique for order preference by similarity to ideal solution (FTOPSIS) approach [30], so as to recommend smart technology applications that are more suitable for supporting mobile healthcare in the later stage of the pandemic. In the literature, fuzzy methods or systems have been applied to choose a suitable recovery plan for an ecotourism center [31], choose suitable lockdown relaxation protocols for a government [32], identify and compare risk factors for the spread of COVID-19 [33], classify countries based on their confirmed, recovered, and deaths cases [34], cluster countries according to the spread rates of COVID-19 in these countries [35], etc. These applications are subject to similar problems: the inaccuracy of data, the divergence of subjective opinions, unknown suitability of existing analysis methods, etc.

The contribution of this study is described as follows. Prior to the COVID-19 pandemic, smart technologies have been widely applied to mobile health care [36]. It will be the same after the pandemic is over. This study does not claim that this trend will change but aims to compare whether the widely used smart technologies are different before and after the COVID-19 pandemic. For this reason, this research applies a fuzzy method to analyze the collected data. The contribution of this research lies in the comparison of the characteristics of smart technologies applied to mobile health care before and after the pandemic. The fuzzy method applied in this study is just a data analysis tool. Other analysis methods, such as stochastic methods, are also applicable.

The rest of this paper is organized as follows. Section 2 analyzes smart technology applications to support mobile healthcare within the COVID-19 pandemic and compares them with those before the pandemic. Based on the comparison results, possible developments in smart technology applications to support mobile healthcare in the later stages of the COVID-19 pandemic are estimated. Section 3 introduces the CFGM-FTOPSIS approach for assessing and comparing possible smart technology applications to support mobile healthcare in the later stage of the pandemic. Section 4 summarizes this study and outlines some topics that may be investigated in the future.

## 2. Past Work

**Figure 1:**
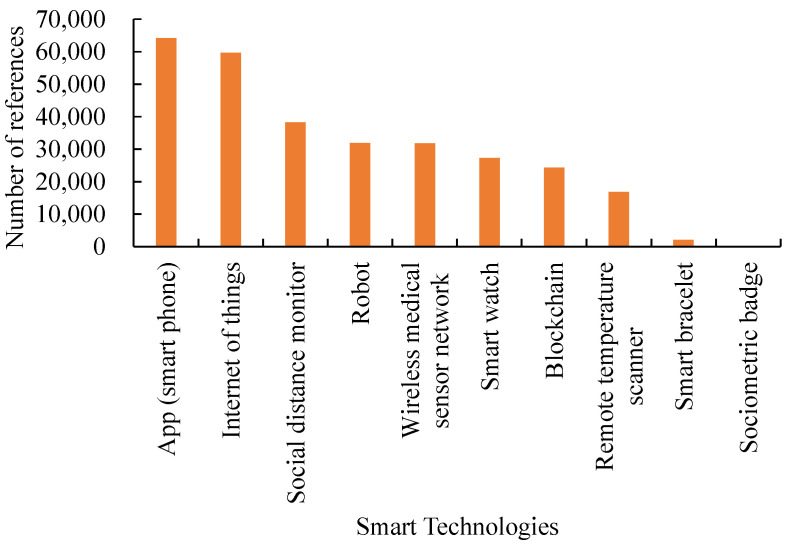
Number of references related to a specific smart technology application to mobile healthcare since 2020 [43].

We reviewed the literature and reports on the applications of smart technologies to mobile healthcare. A summary is provided in Figure 1, showing the number of references related to specific smart technology applications to mobile healthcare since 2020. Some smart technologies, such as blockchain [37,38] and the Internet of things (IoT) [39,40], that are relatively hot in research but not yet popularized [41,42], were excluded from the scope of experimental comparison in this study.

### 2.1. Comparing Smart Technology Applications to Support Mobile Healthcare before and within the COVID-19 Pandemic

Smart technology applied during the COVID-19 pandemic had the following differences from those applied before the outbreak.

First, users’ motivations for applying smart technologies have changed. Before the outbreak of the COVID-19 pandemic, smart technology applications were called for a healthy life. In contrast, during the pandemic, applications are mainly to avoid infection (i.e., the prevention of diseases).

In addition, some innovative ways of applying smart technologies within the COVID-19 pandemic have emerged. Smart technologies are applied both before and within the COVID-19 pandemic. However, during the COVID-19 pandemic, many existing smart technologies have been innovatively applied: For example, apps were designed to help find where to buy facial masks, remind a user to wear a facial mask or detect whether a user is wearing a facial mask [44]. According to the statistics by Collado-Borrell et al. [45], most of the apps developed at this stage were used to provide information or news about COVID-19, record symptoms, and trace contacts. In addition, smart watches were also applied to track people’s health conditions (including heart rate and sleep duration) and physical activities (including gesture, motion, step count and movement). This kind of application was relatively rare for smart watches before the pandemic.

Further, users’ acceptance of smart technology applications has increased. During the epidemic, users’ acceptance of certain smart technology applications has increased significantly. For example, according to the survey by Kim et al. [19], hotel tourists were more satisfied with services (including greetings, delivery of goods, cleaning, etc.) provided by humans than by robots before the COVID-19 pandemic but felt more at ease if these services were provided by robots during the pandemic.

Furthermore, new smart technologies are still being proposed. Some new smart technologies have been developed and applied during the COVID-19 pandemic. For example, a novel smart bracelet, as an application of the Internet of things, was developed and applied during the COVID-19 pandemic to measure the body temperature and blood oxygen level of a patient [20]. In addition, the GPS tracker on the smart bracelet helps ensure the patient’s commitment to quarantine and social distancing. Sociometric badges [21] can also be applied to fulfill similar purposes. These smart technologies were previously used to promote interpersonal communication [22,23,24,25], but now they can be used to monitor and restrict interpersonal interaction. Smart devices like smart bracelets are undoubtedly an important part of a wireless medical sensor network [26]. During the COVID-19 pandemic, a wireless medical sensor network can not only monitor the health conditions of patients ubiquitously but also reduce the contact between doctors (and nurses) and patients.

### 2.2. Possible Developments in Smart Technology Applications to Support Mobile Healthcare after the COVID-19 Pandemic

Some possible developments in smart technology applications to support mobile healthcare in the later stages of (or after) the COVID-19 pandemic is estimated as follows.

First, the motivations for applying smart technologies change again. After the COVID-19 pandemic, an increasingly strong motivation for applying smart technologies is to restore the freedom of mobility. A vaccine passport is an obvious example [27]. Only travelers who have been vaccinated are allowed to enter some countries or regions [29,46]. In addition, to promote safe cross-border travel, websites or apps for booking rooms, seats, or restaurants across countries also consider the requirements for vaccination in tourist destinations.

Second, the demand for some smart technology applications may disappear. For example, after widespread vaccination, the need to avoid human contact with smart technologies, such as robots and drones is no longer strong. In addition, the costs and expenses of widespread deployment of robots are also unbearable. For these reasons, applications of robots and drones may fade out after the pandemic. In addition, in the survey of Sierra Marín et al. [47], some hospital staff were afraid of being replaced by robots.

In addition, the requirements for the effectiveness of a smart technology application will become higher. The performances of some existing smart technology applications are not sufficiently high. For example, according to the experiment of Suhartina and Abuzairi [48], the blood oxygen level detected by a smart bracelet is usually lower than the actual value. The deviation may exceed 5%. In addition, smart bracelets, smart watches, and smart body temperature monitors (i.e., infrared cameras/thermometers) are subject to the same limitation—that is, a patient is contagious two days before the onset of obvious symptoms [49], while pre-symptomatic or asymptomatic patients cause more than half of infections. During the COVID-19 pandemic, most users can tolerate this problem. However, after the pandemic is over, users will have higher requirements for the effectiveness of a smart technology application.

Further, smart technology applications must be enhanced by software developments. For example, to solve the problem that the detection accuracy of a smart bracelet is not high enough, researchers have suggested that a patient must continue to record any symptom accompanied with the detection data of the smart bracelet to conduct a comprehensive judgment using special algorithms [49]. Similarly, a number of studies [50,51,52] also recommended combining smartwatch detection results with self-reported symptoms to determine whether an individual has COVID-19 after symptoms appear.

Furthermore, the cost-effectiveness of a smart technology application will be more rigorously examined. For example, when the pandemic is severe, managing the massive amounts of heterogeneous data [53] transmitted through a wireless medical sensor network requires huge hardware investment [26], which limits its application to a small, experimental space. For this reason, after the COVID-19 pandemic, the reliance on wireless medical sensor networks will be greatly reduced. In addition, compared with smart bracelets, smart watches are equipped with more sensors, so they are more powerful in tracking people’s health conditions and physical activities. However, smart watches are usually more expensive than smart bracelets. Nevertheless, smart watches are more sophisticated than smart bracelets and are expected to be continuously used after the COVID-19 pandemic. Further, since high body temperature is one of the signs of COVID-19, remote temperature scanners have been widely applied to screen people who may be infectious [54]. Although the accuracy of this is not high [55], this technology is quick, simple, mature and non-invasive, and can prevent the spread of other diseases. Therefore, after the pandemic is over, it is expected to be continuously applied.

Table 1 summarizes the discussion result. It can be seen from this table that the motivations for applying smart technologies have changed from promoting a healthy life before the COVID-19 outbreak to avoiding infection during the pandemic. After the COVID-19 pandemic is over, the motivation is to promote healthy mobility.

## 3. Assessing Smart Technology Applications after the COVID-19 Pandemic

**Figure 2:**
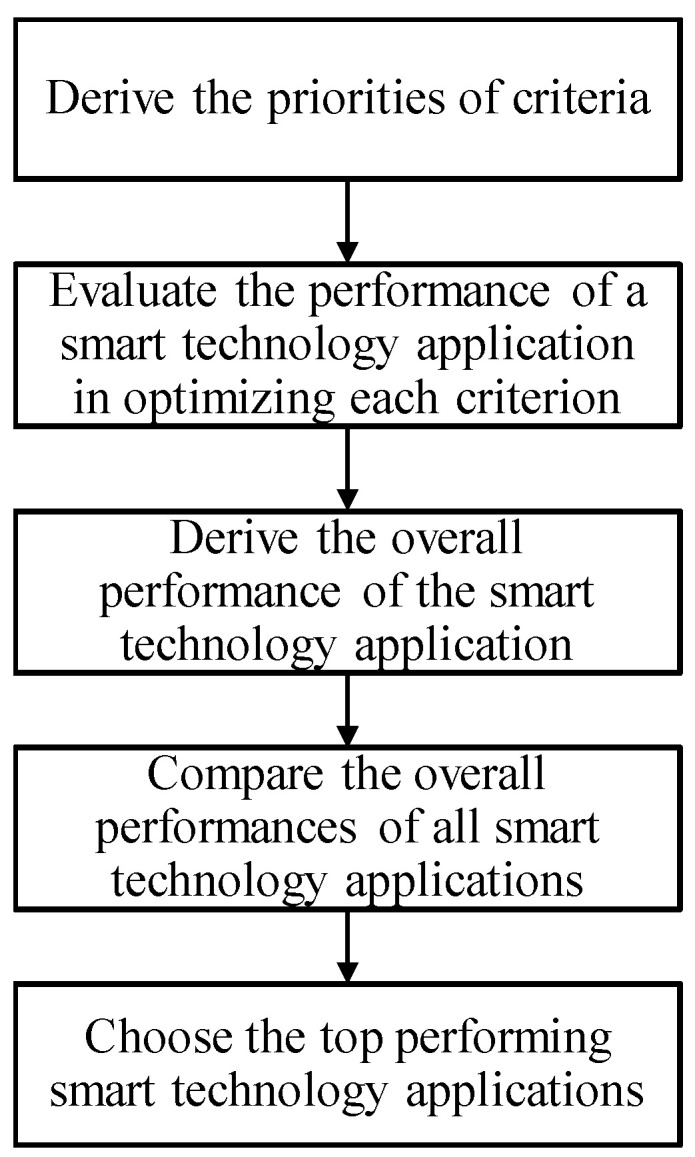
Procedure for the CFGM-FTOPSIS approach.

Based on the above discussion, the CFGM-FTOPSIS [30] is applied to assess a smart technology application to support mobile healthcare after the COVID-19 pandemic. A fuzzy approach is suitable for considering the many uncertainties that people face during the COVID-19 pandemic. Without loss of generality, all fuzzy parameters and variables in the proposed methodology are given in or approximated by triangular fuzzy numbers (TFNs). The procedure for the fuzzy MCDM approach is illustrated in Figure 2. The first step is to derive the fuzzy priorities of criteria for assessing a smart technology application.

In the CFGM-FTOPSIS approach, first, the decision-maker expresses his/her opinion on the relative priority of a criterion over another in linguistic (or semantic) terms. These linguistic terms are usually mapped to TFNs within [1,9,56] (see Table 2). The results are summarized with a fuzzy judgment matrix $\tilde{A}=[{\tilde{a}}_{ij}]$; *i*, *j* = 1 ~ *n*. ${\tilde{a}}_{ij}$ is the relative priority of criterion *i* over criterion *j*. The TFNs for these linguistic terms overlap to embody the uncertainty in choosing between them.

### 3.1. Deriving the Fuzzy Priorities of Criteria Using CFGM

The fuzzy judgment matrix meets the following conditions:

(1) $$det(\tilde{A}(-)\tilde{\lambda }I)=0$$

(2) $$(\tilde{A}(-)\tilde{\lambda }I)(\times )\tilde{x}=0$$

$\tilde{\lambda }$ is the fuzzy maximal eigenvalue; $\tilde{x}$ is the fuzzy eigenvector. (−) and (×) denote fuzzy subtraction and multiplication [58], respectively. The fuzzy priorities of criteria, indicated with $\left\{{\tilde{w}}_{i}|i=1\sim n\right\}$, can be approximated using fuzzy geometric mean (FGM) [56] as:

(3) $$w_{i1}≅\frac{1}{1+\sum_{m\neq i}\frac{\sqrt[{n}]{\prod_{j=1}^{n}a_{mj3}}}{\sqrt[{n}]{\prod_{j=1}^{n}a_{ij1}}}}$$

(4) $$w_{i2}≅\frac{1}{1+\sum_{m\neq i}\frac{\sqrt[{n}]{\prod_{j=1}^{n}a_{mj2}}}{\sqrt[{n}]{\prod_{j=1}^{n}a_{ij2}}}}$$

(5) $$w_{i3}≅\frac{1}{1+\sum_{m\neq i}\frac{\sqrt[{n}]{\prod_{j=1}^{n}a_{mj1}}}{\sqrt[{n}]{\prod_{j=1}^{n}a_{ij3}}}}$$

To improve the approximation precision, Chen and Wang [30] proposed the CFGM approach to tune the values of fuzzy priorities:

(6) $$w_{i1}≅\frac{1}{1+\sum_{m\neq i}\frac{\sqrt[{n}]{\prod_{j=1}^{n}a_{mj3}}}{\sqrt[{n}]{\prod_{j=1}^{n}a_{ij1}}}}+w_{i}^{\left(c\right)}-\frac{1}{1+\sum_{m\neq i}\frac{\sqrt[{n}]{\prod_{j=1}^{n}a_{mj2}}}{\sqrt[{n}]{\prod_{j=1}^{n}a_{ij2}}}}$$

(7) $$w_{i2}≅w_{i}^{\left(c\right)}$$

(8) $$w_{i3}≅\frac{1}{1+\sum_{m\neq i}\frac{\sqrt[{n}]{\prod_{j=1}^{n}a_{mj1}}}{\sqrt[{n}]{\prod_{j=1}^{n}a_{ij3}}}}+w_{i}^{\left(c\right)}-\frac{1}{1+\sum_{m\neq i}\frac{\sqrt[{n}]{\prod_{j=1}^{n}a_{mj2}}}{\sqrt[{n}]{\prod_{j=1}^{n}a_{ij2}}}}$$

where $w_{i}^{\left(c\right)}$ is the (crisp) priority of criterion *i* derived from the defuzzified judgment matrix using an eigen analysis:

(9) $$det(A_{2}-\lambda_{2}I)=0$$

(10) $$(A_{2}-\lambda_{2}I)x_{2}=0$$

Subsequently, ${\tilde{\lambda }}_{\max}$ can be estimated as:

(11) $$\lambda_{\max,1}≅1+\frac{1}{n}\sum_{i=1}^{n}\sum_{j\neq i}\frac{a_{ij1}w_{j1}}{w_{i3}}$$

(12) $$\lambda_{\max,2}≅1+\frac{1}{n}\sum_{i=1}^{n}\sum_{j\neq i}\frac{a_{ij2}w_{j2}}{w_{i2}}$$

(13) $$\lambda_{\max,3}≅1+\frac{1}{n}\sum_{i=1}^{n}\sum_{j\neq i}\frac{a_{ij3}w_{j3}}{w_{i1}}$$

$\tilde{A}$ is consistent if:

(14) $$\tilde{CR}(\tilde{A})\leq 0.1$$

where:

(15) $$CR_{1}(\tilde{A})=\max(0,\;\frac{\lambda_{\max,1}-n}{(n-1)RI})$$

(16) $$CR_{2}(\tilde{A})=\frac{\lambda_{\max,2}-n}{(n-1)RI}$$

(17) $$CR_{3}(\tilde{A})=\frac{\lambda_{\max,3}-n}{(n-1)RI}$$

*RI* denotes random consistency index [59]. It is noteworthy that $CR_{1}(\tilde{A})$ is not necessarily associated with $\lambda_{\max,1}$, while $CR_{3}(\tilde{A})$ is usually determined by $\lambda_{\max,3}$. However, $CR_{3}(\tilde{A})$ considers the most inconsistent case that rarely happens. Therefore, in practice, it is better that $CR_{2}(\tilde{A})\leq 0.1\sim 0.3$ and $CR_{1}(\tilde{A})=0$. Otherwise, the decision maker needs to modify his/her pairwise comparison results.

### 3.2. Assessing a Smart Technology Application Using FTOPSIS

Letting the performance of smart technology application *q* in optimize criterion *i* be indicated with ${\tilde{p}}_{qi}$. To facilitate the aggregation, ${\tilde{p}}_{qi}$ is normalized as [60,61]:

(18) $$\begin{array}{cc} {\tilde{\rho }}_{qi} & =\frac{{\tilde{p}}_{qi}}{\sqrt{\sum_{\phi =1}^{Q}{\tilde{p}}_{\phi i}^{2}}}\\ & =\frac{1}{\sqrt{1+\sum_{\phi \neq q}{\left(\frac{{\tilde{p}}_{\phi i}}{{\tilde{p}}_{qi}}\right)}^{2}}} \end{array}$$

After expansion,

(19) $$\rho_{qi1}=\frac{1}{\sqrt{1+\sum_{\phi \neq q}{\left(\frac{p_{\phi i3}}{p_{qi1}}\right)}^{2}}}$$

(20) $$\rho_{qi2}=\frac{1}{\sqrt{1+\sum_{\phi \neq q}{\left(\frac{p_{\phi i2}}{p_{qi2}}\right)}^{2}}}$$

(21) $$\rho_{qi3}=\frac{1}{\sqrt{1+\sum_{\phi \neq q}{\left(\frac{p_{\phi i1}}{p_{qi3}}\right)}^{2}}}$$

The fuzzy priority of the criterion is multiplied by the normalized performance to derive the fuzzy prioritized score as:

(22) $${\tilde{s}}_{qi}={\tilde{w}}_{i}(\times ){\tilde{\rho }}_{qi}$$

Equivalently,

(23) $$s_{qi1}=w_{i1}\rho_{qi1}$$

(24) $$s_{qi2}=w_{i2}\rho_{qi2}$$

(25) $$s_{qi3}=w_{i3}\rho_{qi3}$$

Then, two reference points representing the theoretically best and worst alternatives are defined as ${\tilde{\Lambda }}_{i}^{+}$ and ${\tilde{\Lambda }}_{i}^{-}$, respectively:

(26) $$\begin{array}{cc} {\tilde{\Lambda }}_{i}^{+} & =\underset{q}{\max}{\tilde{s}}_{qi}\\ & ≅(\underset{q}{\max}s_{qi1},\;\underset{q}{\max}s_{qi2},\;\underset{q}{\max}s_{qi3}) \end{array}$$

(27) $$\begin{array}{cc} {\tilde{\Lambda }}_{i}^{-} & =\underset{q}{\min}{\tilde{s}}_{qi}\\ & ≅(\underset{q}{\min}s_{qi1},\;\underset{q}{\min}s_{qi2},\;\underset{q}{\min}s_{qi3}) \end{array}$$

The overall performance of a smart technology application is evaluated by comparing its (Euclidean) distances to the two reference points:

(28) $$\begin{array}{cc} {\tilde{U}}_{q} & =\frac{{\tilde{d}}_{q}^{-}}{{\tilde{d}}_{q}^{+}(+){\tilde{d}}_{q}^{-}}\\ & =(\frac{1}{\frac{d_{q3}^{+}}{d_{q1}^{-}}+1},\;\frac{1}{\frac{d_{q2}^{+}}{d_{q2}^{-}}+1},\;\frac{1}{\frac{d_{q1}^{+}}{d_{q3}^{-}}+1}) \end{array}$$

where:

(29) $$\begin{array}{cc} {\tilde{d}}_{q}^{+} & =\sqrt{\sum_{i=1}^{n}{\left({\tilde{\Lambda }}_{i}^{+}(-){\tilde{s}}_{qi}\right)}^{2}}\\ & =(\sqrt{\sum_{i=1}^{n}\max{\left(\Lambda_{i1}^{+}-s_{qi3},\;0\right)}^{2}},\;\sqrt{\sum_{i=1}^{n}{\left(\Lambda_{i2}^{+}-s_{qi2}\right)}^{2}},\;\sqrt{\sum_{i=1}^{n}{\left(\Lambda_{i3}^{+}-s_{qi1}\right)}^{2}}) \end{array}$$

(30) $$\begin{array}{cc} {\tilde{d}}_{q}^{-} & =\sqrt{\sum_{i=1}^{n}{\left({\tilde{s}}_{qi}(-){\tilde{\Lambda }}_{i}^{-}\right)}^{2}}\\ & =(\sqrt{\sum_{i=1}^{n}\max{\left(s_{qi1}-\Lambda_{i3}^{-},\;0\right)}^{2}},\;\sqrt{\sum_{i=1}^{n}{\left(s_{qi2}-\Lambda_{i2}^{-}\right)}^{2}},\;\sqrt{\sum_{i=1}^{n}{\left(s_{qi3}-\Lambda_{i1}^{-}\right)}^{2}}) \end{array}$$

## 4. Case Study

### Background

The proposed methodology has been applied to assess and compare several potential smart technology applications for supporting mobile healthcare after the COVID-19 pandemic from the literature and reports.

The decision-makers were the members of a one-year project entitled “Smart Technology Applications for Supporting Medical and Health Care after the COVID-19 Pandemic”, including an industrial engineering professor, an information technology engineer, and a healthcare technology researcher.

From the discussion in Section 3, they listed all possible factors that should be considered. Then, through brainstorming, the less influential factors were deleted one by one. Finally, the following factors were considered critical when assessing a smart technology application for supporting mobile healthcare after the COVID-19 pandemic:

- Can it provide value-added services based on vaccination information [27,28,29]?
- Is it cost effective [20,26,43,44]?
- Can it enhance people’s mobility healthily [18,29]?
- Is it necessary or irreplaceable [18,41,42]?
- Can it be combined with other smart technology applications to achieve synergy [20,26]?
- Is it easy to implement and maintain [41,42,54]?

The relative priorities of these critical factors were compared in pairs. After discussion, the three decision-makers jointly selected a linguistic variable representing the relative priority. The results are summarized in Table 3.

Based on Table 3, the following fuzzy judgment matrix was constructed:

$$\tilde{A}=\left[\begin{array}{cccccc} 1 & \left(1,\;2,\;4\right) & - & \left(1,\;3,\;5\right) & \left(1,\;3,\;5\right) & \left(3,\;5,\;7\right)\\ - & 1 & - & \left(2,\;4,\;6\right) & \left(3,\;5,\;7\right) & \left(2,\;4,\;6\right)\\ \left(2,\;4,\;6\right) & \left(3,\;5,\;7\right) & 1 & (3,\;5,\;7) & \left(3,\;5,\;7\right) & \left(3,\;5,\;7\right)\\ - & - & - & 1 & \left(3,\;5,\;7\right) & \left(3,\;5,\;7\right)\\ - & - & - & - & 1 & -\\ - & - & - & - & \left(1,\;2,\;4\right) & 1 \end{array}\right]$$

$\tilde{CR}(\tilde{A})=(0.00,\;0.13,\;6.16)$, which was considered sufficiently consistent considering the uncertainty of the problem under investigation.

**Figure 3:**
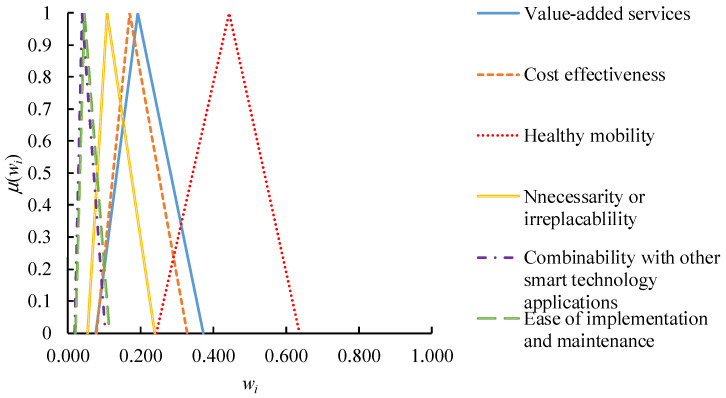
Procedure for the CFGM-FTOPSIS approach.

To derive the fuzzy priorities of criteria from the fuzzy judgment matrix, the CFGM approach was applied. The CFGM approach was implemented using Excel on a PC with an i7-7700 CPU of 3.6 GHz and 8 GB of RAM (ASUS, Hsinchu City, Taiwan). The results are shown in Figure 3. Obviously, “healthy mobility” was more important than the others, followed by “value-added services”

Based on the derived fuzzy priorities of criteria, nine potential smart technology applications (see Table 4) were assessed and compared using FTOPSIS [20,54,55]. References that support these smart technology applications are also provided. These smart technology applications were enumerated and selected by project members after brainstorming. Therefore, this list did not cover all possible smart technology applications to mobile healthcare. In other studies, the smart technology applications chosen by different decision-makers will be different.

The criteria for evaluating the performances of these smart technology applications have been established, as summarized in Table 5. All performances are normalized into [1,5] to facilitate the subsequent aggregation. The evaluation results are summarized in Table 6.

First, the performance of a smart technology application in optimizing each criterion was normalized using fuzzy distributive normalization. The results are summarized in Table A1 of Appendix A.

After multiplying the derived fuzzy priorities to the normalized performances, the fuzzy prioritized scores of smart technology applications were obtained. The results are summarized in Table A2 of Appendix A.

Based on the fuzzy prioritized scores of all smart technology applications, the fuzzy ideal point and the fuzzy anti-ideal point were respectively defined in Table A3 of Appendix A. Subsequently, the distances from each smart technology application, to the two reference points were measured respectively. The results are summarized in Table A4 of Appendix A. A smart technology application was better if it was closer to the ideal solution but farther from the anti-ideal solution.

Finally, the overall performance of each smart technology application, in terms of its fuzzy closeness, was evaluated. The results are shown in Table 7. The overall performance was also defuzzified using the center-of-gravity (COG) method [64]. Based on the defuzzification results, the smart technology applications were ranked. 

According to the experimental results, the following discussion was made:

(1) “Vaccine passport and related applications” was the best smart technology application to support mobile healthcare after the COVID-19 pandemic, followed by “smart watches” and “contact tracing apps”.
(2) For comparison, the prevalent FGM method [55] was applied to this case. The fuzzy priorities of criteria were derived as:
  $${\tilde{w}}_{1}=\left(0.084,\;0.201,\;0.381\right)$$
  $${\tilde{w}}_{2}=\left(0.078,\;0.169,\;0.326\right)$$
  $${\tilde{w}}_{3}=\left(0.240,\;0.440,\;0.633\right)$$
  $${\tilde{w}}_{4}=\left(0.051,\;0.103,\;0.236\right)$$
  $${\tilde{w}}_{5}=\left(0.019,\;0.040,\;0.104\right)$$
  $${\tilde{w}}_{6}=\left(0.023,\;0.048,\;0.116\right)$$
  Subsequently, fuzzy weighted average (FWA) was applied to evaluate the overall performance of a smart technology application to support mobile healthcare after the COVID-19 pandemic. The evaluation results are summarized in Table 8. The top two smart technology applications were also “vaccine passport and related applications” and “smart watches”. However, the ranking results of other smart technology applications were slightly different from those obtained using the proposed methodology.
(3) In addition, the fuzzy extent analysis (FEA) method proposed by Chang [65] was also applied to this case. The priorities of criteria derived using FEA were crisp values. Based on the derived priorities, the overall performance of a smart technology application was evaluated using FWA. The evaluation results are presented in Table 9. “Vaccine passport and related applications” and “smart watches” outperformed the other smart technology applications. In addition, the ranking results of smart technology applications were somewhat different from those obtained using the previous two methods.
(4) If five smart technology applications were to be selected, the results using various methods are compared in Table 10. The same smart technology applications were recommended, showing the robustness of the evaluation results.

## 5. Discussion and Conclusions

Applications of smart technologies have widely improved mobile healthcare before the COVID-19 pandemic, and it will be the same after the pandemic. However, smart technology applications for supporting mobile healthcare after the COVID-19 pandemic may not be the same as those before the pandemic. To address this issue, this study compares smart technology applications to support mobile healthcare within the COVID-19 pandemic with those before the pandemic, so as to estimate possible developments in smart technology applications after the COVID-19 pandemic. In addition, to quantitatively assess and compare possible smart technology applications to support mobile healthcare after the COVID-19 pandemic, the CFGM-FTOPSIS approach is applied.

The effectiveness of the proposed methodology was validated by applying it to nine possible smart technology applications to support mobile healthcare after the COVID-19 pandemic. Two existing fuzzy MCDM methods were also applied to these smart technology applications for comparison. According to the experimental results, the following conclusions were made:

“Vaccine passport and related applications” and “smart watches” were the best smart technology applications to support mobile healthcare after the COVID-19 pandemic. These two smart technology applications are already mature, which may be the main reason for their preference. The top five smart technology applications included “contact tracing apps”, “smart bracelets”, and “wireless medical sensor networks”. These smart technology applications have a common feature, that is, they can (or have the potential) to support a large number of users.

(1) In contrast, although remote body temperature monitoring was a very prevalent smart technology application, it was not recommended. The reason should be limited functions and insufficient effectiveness. At the peak of the COVID-19 pandemic, people were more tolerant of these shortcomings.
(2) Except for the two top performers, the rankings of other smart technologies were different from those calculated using existing methods. Such differences resulted from errors in estimating the priorities of critical factors. Taking FEA as an example; it overestimated the priorities of “cost effectiveness” and “necessity or irreplaceability” but underestimated the priority of “healthy mobility”. As a result, the ranking of “contact tracing app” fell to the fourth. The CFGM method applied in this study solved this problem in an efficient and effective way.
(3) Nevertheless, if five smart technology applications were to be recommended, the results using various methods were the same, supporting the robustness of the recommendation results.

This study has the following limitations:

(1) Although the critical factors considered in this study referred to related literature or reports, these critical factors were subjectively selected by the researchers. Critical factors considered by other researchers will not be the same.
(2) In addition, the relative priorities of critical factors were subjectively determined by the decision makers and are susceptible to personal bias. If more decision makers can be involved to make a joint decision, subjective bias can be avoided.

Hopefully, the conclusions drawn in this study will be examined in the near future. In addition, there is still considerable room for the development and application of smart technologies in this field.

## Figures and Tables

**Table 1:** Summary of the discussion result.

Characteristics	Before COVID-19	Within COVID-19	After COVID-19
Motivations	Healthy life	Avoidance of infection	Restoring mobility
Users’ Acceptance	Low~Moderate	Very high	High
Increasingly Applied Smart Technologies	Smart watches Restaurant recommendation apps (for healthy diets) (Smart phones) Apps for outdoor exercise or fitness	Robots (or drones) Smart body temperature monitors Smart bracelets Smart watches	Smart body temperature monitors Smart watches (Smart phones) vaccine passports
Decreasingly Applied Smart Technologies	Robots (or drones) Smart body temperature monitors Smart bracelets Wireless medical sensor network	Restaurant recommendation apps (for healthy diets) (Smart phones) Apps for outdoor exercise or fitness Wireless medical sensor network	Robots (or drones) Wireless medical sensor network
Performance	Balanced with cost effectiveness	As high as possible Not critical	Critical
Cost Effectiveness	Balanced with performance	As high as possible Not critical	Rigorously examined

**Table 2:** TFNs for expressing linguistic terms *.

Linguistic Term	TFN
As equal as	(1, 1, 3)
As equal as or weakly more important than	(1, 2, 4)
Weakly more important than	(1, 3, 5)
Weakly or strongly more important than	(2, 4, 6)
Strongly more important than	(3, 5, 7)
Strongly or very strongly more important than	(4, 6, 8)
Very strongly more important than	(5, 7, 9)
Very strongly or absolutely more important than	(6, 8, 9)
Absolutely more important than	(7, 9, 9)

*: Adapted/Reprinted from [57].

**Table 3:** Pairwise comparison results.

Critical Factor I	Relative Priority	Critical Factor II
Value-added services	As equal as or weakly more important than	Cost effectiveness
Healthy mobility	Weakly or strongly more important than	Value-added services
Value-added services	Weakly more important than	Necessity or irreplaceability
Value-added services	Weakly more important than	Combinability with other smart technology applications
Value-added services	Strongly more important than	Ease of implementation and maintenance
Healthy mobility	Strongly more important than	Cost effectiveness
Cost effectiveness	Weakly or strongly more important than	Necessity or irreplaceability
Cost effectiveness	Strongly more important than	Combinability with other smart technology applications
Cost effectiveness	Weakly or strongly more important than	Ease of implementation and maintenance
Healthy mobility	Strongly more important than	Necessity or irreplaceability
Healthy mobility	Strongly more important than	Combinability with other smart technology applications
Healthy mobility	Strongly more important than	Ease of implementation and maintenance
Necessity or irreplaceability	Strongly more important than	Combinability with other smart technology applications
Necessity or irreplaceability	Strongly more important than	Ease of implementation and maintenance
Ease of implementation and maintenance	As equal as or weakly more important than	Combinability with other smart technology applications

**Table 4:** Potential smart technology applications for supporting mobile healthcare after the COVID-19 pandemic.

*q*	Smart Technology Application	Source
1	Vaccine passport and related apps	Hall and Studdert [27], Wu et al. [46]
2	Sterilization robot	Nichols [14]
3	Smart bracelet (for monitoring body temperature and blood oxygen level)	Ennafiri and Mazri [20], Suhartina and Abuzairi [48]
4	Smart watch (for monitoring body temperature, blood oxygen level, heart rate, sleep duration, gesture, motion, step count, and movement).	Mishra et al. [50], Zhu et al. [27], Niela-Vilén et al. [52]
5	Social distance monitor	Bian et al. [15]
6	Remote temperature scanner	Inn [50], Tipton and Mekjavic [55]
7	Wireless medical sensor network	Ali et al. [26]
8	Healthcare robot (for monitoring patient’s physiological conditions, supporting surgery, dispensing medication, assisting patients with cognition challenges and disabilities, etc.)	Kaiser et al. [62]
9	Contact tracing app	Abbas and Michael [63]

**Table 5:** Criteria for evaluating the performance of a smart technology application.

Critical Feature	Criterion
Value-added services	${\tilde{p}}_{q1}(x_{q1})=\{\begin{array}{ccc} \left(0,\;0,\;1\right) & \mathrm{if} & x_{q1}=“\mathrm{Very}\;\mathrm{Low}\;\mathrm{Value-added}”\;\mathrm{or}\;\mathrm{data}\;\mathrm{not}\;\mathrm{available}\\ \left(0,\;1,\;2\right) & \mathrm{if} & x_{q1}=“\mathrm{Low}\;\mathrm{Value-added}”\\ \left(1.5,\;2.5,\;3.5\right) & \mathrm{if} & x_{q1}=“\mathrm{Moderately}\;\mathrm{Value-added}”\\ \left(3,\;4,\;5\right) & \mathrm{if} & x_{q1}=“\mathrm{High}\;\mathrm{Value-added}”\\ \left(4,\;5,\;5\right) & \mathrm{if} & x_{q1}=“\mathrm{Very}\;\mathrm{High}\;\mathrm{Value-added}” \end{array}$ where $x_{q1}$ is the services provided by the *q*-th smart technology application.
Cost effectiveness	${\tilde{p}}_{q1}(x_{q1})=\{\begin{array}{ccc} \left(0,\;0,\;1\right) & \mathrm{if} & 0.1\;\underset{r}{\min}\;x_{r2}+0.9\;\underset{r}{\max}\;x_{r2}\leq x_{q2}\;\mathrm{or}\;\mathrm{data}\;\mathrm{not}\;\mathrm{available}\\ \left(0,\;1,\;2\right) & \mathrm{if} & 0.35\;\underset{r}{\min}\;x_{r2}+0.65\;\underset{r}{\max}\;x_{r2}\leq x_{q2}<0.1\;\underset{r}{\min}\;x_{r2}+0.9\;\underset{r}{\max}\;x_{r2}\\ \left(1.5,\;2.5,\;3.5\right) & \mathrm{if} & 0.65\;\underset{r}{\min}\;x_{r2}+0.35\;\underset{r}{\max}\;x_{r2}\leq x_{q2}<0.35\;\underset{r}{\min}\;x_{r2}+0.65\;\underset{r}{\max}\;x_{r2}\\ \left(3,\;4,\;5\right) & \mathrm{if} & 0.9\;\underset{r}{\min}\;x_{r2}+0.1\;\underset{r}{\max}\;x_{r2}\leq x_{q2}<0.65\;\underset{r}{\min}\;x_{r2}+0.35\;\underset{r}{\max}\;x_{r2}\\ \left(4,\;5,\;5\right) & \mathrm{if} & x_{q2}<0.9\;\underset{r}{\min}\;x_{r2}+0.1\;\underset{r}{\max}\;x_{r2} \end{array}$ where $x_{q2}$ is the total costs of the *q*-th smart technology application.
Healthy mobility	${\tilde{p}}_{q3}(x_{q3})=\{\begin{array}{ccc} \left(0,\;0,\;1\right) & \mathrm{if} & x_{q3}=“\mathrm{Very}\;\mathrm{Low}”\;\mathrm{or}\;\mathrm{data}\;\mathrm{not}\;\mathrm{available}\\ \left(0,\;1,\;2\right) & \mathrm{if} & x_{q3}=“\mathrm{Low}”\\ \left(1.5,\;2.5,\;3.5\right) & \mathrm{if} & x_{q3}=“\mathrm{Moderate}”\\ \left(3,\;4,\;5\right) & \mathrm{if} & x_{q3}=“\mathrm{High}”\\ \left(4,\;5,\;5\right) & \mathrm{if} & x_{q3}=“\mathrm{Very}\;\mathrm{High}” \end{array}$ where $x_{q3}$ is the degree to which healthy mobility can be improved by the *q*-th smart technology application.
Necessity or irreplaceability	${\tilde{p}}_{q4}(x_{q4})=\{\begin{array}{ccc} \left(0,\;0,\;1\right) & \mathrm{if} & x_{q4}=“\mathrm{Very}\;\mathrm{Low}”\;\mathrm{or}\;\mathrm{data}\;\mathrm{not}\;\mathrm{available}\\ \left(0,\;1,\;2\right) & \mathrm{if} & x_{q4}=“\mathrm{Low}”\\ \left(1.5,\;2.5,\;3.5\right) & \mathrm{if} & x_{q4}=“\mathrm{Moderate}”\\ \left(3,\;4,\;5\right) & \mathrm{if} & x_{q4}=“\mathrm{High}”\\ \left(4,\;5,\;5\right) & \mathrm{if} & x_{q4}=“\mathrm{Very}\;\mathrm{High}” \end{array}$ where $x_{q4}$ is the necessity or irreplaceability of the *q*-th smart technology application.
Combinability with other smart technology applications	${\tilde{p}}_{q5}(x_{q5})=\{\begin{array}{ccc} \left(0,\;0,\;1\right) & \mathrm{if} & x_{q5}=“\mathrm{Very}\;\mathrm{Low}”\;\mathrm{or}\;\mathrm{data}\;\mathrm{not}\;\mathrm{available}\\ \left(0,\;1,\;2\right) & \mathrm{if} & x_{q5}=“\mathrm{Low}”\\ \left(1.5,\;2.5,\;3.5\right) & \mathrm{if} & x_{q5}=“\mathrm{Moderate}”\\ \left(3,\;4,\;5\right) & \mathrm{if} & x_{q5}=“\mathrm{High}”\\ \left(4,\;5,\;5\right) & \mathrm{if} & x_{q5}=“\mathrm{Very}\;\mathrm{High}” \end{array}$ where $x_{q5}$ is the combinability of the *q*-th smart technology application with other smart technology applications.
Ease of implementation and maintenance	${\tilde{p}}_{q6}(x_{q6})=\{\begin{array}{ccc} \left(0,\;0,\;1\right) & \mathrm{if} & x_{q6}=“\mathrm{Very}\;\mathrm{Easy}”\;\mathrm{or}\;\mathrm{data}\;\mathrm{not}\;\mathrm{available}\\ \left(0,\;1,\;2\right) & \mathrm{if} & x_{q6}=“\mathrm{Easy}”\\ \left(1.5,\;2.5,\;3.5\right) & \mathrm{if} & x_{q6}=“\mathrm{Moderate}”\\ \left(3,\;4,\;5\right) & \mathrm{if} & x_{q6}=“\mathrm{Difficult}”\\ \left(4,\;5,\;5\right) & \mathrm{if} & x_{q6}=“\mathrm{Very}\;\mathrm{Difficult}” \end{array}$ where $x_{q6}$ is the ease of implementation and maintenance of the *q*-th smart technology application.

**Table 6:** Performances of smart technology applications.

*q*	${\tilde{p}}_{q1}$	${\tilde{p}}_{q2}$	${\tilde{p}}_{q3}$	${\tilde{p}}_{q4}$	${\tilde{p}}_{q5}$	${\tilde{p}}_{q6}$
1	(3, 4, 5)	(1.5, 2.5, 3.5)	(4, 5, 5)	(3, 4, 5)	(1.5, 2.5, 3.5)	(1.5, 2.5, 3.5)
2	(1.5, 2.5, 3.5)	(0, 0, 1)	(1.5, 2.5, 3.5)	(1.5, 2.5, 3.5)	(3, 4, 5)	(1.5, 2.5, 3.5)
3	(1.5, 2.5, 3.5)	(1.5, 2.5, 3.5)	(3, 4, 5)	(1.5, 2.5, 3.5)	(1.5, 2.5, 3.5)	(3, 4, 5)
4	(3, 4, 5)	(1.5, 2.5, 3.5)	(3, 4, 5)	(1.5, 2.5, 3.5)	(3, 4, 5)	(1.5, 2.5, 3.5)
5	(0, 1, 2)	(3, 4, 5)	(1.5, 2.5, 3.5)	(1.5, 2.5, 3.5)	(3, 4, 5)	(3, 4, 5)
6	(0, 1, 2)	(1.5, 2.5, 3.5)	(1.5, 2.5, 3.5)	(0, 1, 2)	(0, 1, 2)	(4, 5, 5)
7	(4, 5, 5)	(0, 1, 2)	(3, 4, 5)	(1.5, 2.5, 3.5)	(1.5, 2.5, 3.5)	(0, 1, 2)
8	(1.5, 2.5, 3.5)	(0, 0, 1)	(3, 4, 5)	(0, 1, 2)	(1.5, 2.5, 3.5)	(1.5, 2.5, 3.5)
9	(3, 4, 5)	(1.5, 2.5, 3.5)	(3, 4, 5)	(0, 1, 2)	(1.5, 2.5, 3.5)	(3, 4, 5)

**Table 7:** Overall performances of smart technology applications.

*q*	${\tilde{C}}_{q}$	Defuzzified Value	Rank
1	(0, 0.76, 1)	0.587	1
2	(0, 0.21, 1)	0.404	9
3	(0, 0.55, 1)	0.517	4
4	(0, 0.63, 1)	0.544	2
5	(0, 0.44, 1)	0.481	6
6	(0, 0.31, 1)	0.438	8
7	(0, 0.54, 1)	0.514	5
8	(0, 0.35, 1)	0.449	7
9	(0, 0.59, 1)	0.529	3

**Table 8:** Overall performances of smart technology applications evaluated using FGM-FWA.

*q*	Overall Performance	Defuzzified Value	Rank
1	(2.37, 4.06, 4.87)	3.76	1
2	(0.88, 2.14, 3.47)	2.16	8
3	(1.8, 3.23, 4.64)	3.23	4
4	(2, 3.52, 4.82)	3.45	2
5	(1.01, 2.58, 4.25)	2.61	6, 7
6	(0.54, 2.1, 3.45)	2.03	9
7	(1.39, 3.34, 4.65)	3.13	5
8	(0.98, 2.58, 4.26)	2.61	6, 7
9	(1.67, 3.38, 4.77)	3.27	3

**Table 9:** Ranking results using FEA-FWA.

*q*	Overall Performance	Defuzzified Value	Rank
1	(3.04, 4.04, 4.66)	3.91	1
2	(1.16, 1.94, 2.94)	2.01	8
3	(2.07, 3.07, 4.07)	3.07	5
4	(2.4, 3.4, 4.4)	3.40	2
5	(1.5, 2.5, 3.5)	2.50	6
6	(0.91, 1.91, 2.91)	1.91	9
7	(2.29, 3.29, 4.07)	3.22	3
8	(1.47, 2.25, 3.25)	2.32	7
9	(2.15, 3.15, 4.15)	3.15	4

**Table 10:** Five smart technology applications were selected using various methods.

Method	Choices
Fuzzy geometric mean (FGM)-Fuzzy weighted average (FWA)	● Vaccine passport and related applications ● Smart watch ● Contact tracing app ● Smart bracelet ● Wireless medical sensor network
Fuzzy extent analysis (FEA)-Fuzzy weighted average (FWA)	● Vaccine passport and related applications ● Smart watch ● Wireless medical sensor network ● Contact tracing app ● Smart bracelet
The proposed methodology	● Vaccine passport and related application ● Smart watches ● Contact tracing app ● Smart bracelet ● Wireless medical sensor network

## Data Availability

Data is contained within the article or Appendix A.

## Appendix A

**Table A1:** Normalized performances of smart technology applications.

*q*	${\tilde{\rho }}_{q1}$	${\tilde{\rho }}_{q2}$	${\tilde{\rho }}_{q3}$	${\tilde{\rho }}_{q4}$	${\tilde{\rho }}_{q5}$	${\tilde{\rho }}_{q6}$
1	(0.26, 0.41, 0.62)	(0.17, 0.36, 0.64)	(0.3, 0.45, 0.57)	(0.33, 0.56, 0.83)	(0.13, 0.28, 0.5)	(0.13, 0.25, 0.44)
2	(0.13, 0.26, 0.45)	(0, 0, 0.22)	(0.11, 0.22, 0.4)	(0.16, 0.35, 0.64)	(0.27, 0.45, 0.68)	(0.13, 0.25, 0.44)
3	(0.13, 0.26, 0.45)	(0.17, 0.36, 0.64)	(0.23, 0.36, 0.55)	(0.16, 0.35, 0.64)	(0.13, 0.28, 0.5)	(0.26, 0.4, 0.61)
4	(0.26, 0.41, 0.62)	(0.17, 0.36, 0.64)	(0.23, 0.36, 0.55)	(0.16, 0.35, 0.64)	(0.27, 0.45, 0.68)	(0.13, 0.25, 0.44)
5	(0, 0.1, 0.27)	(0.34, 0.58, 0.83)	(0.11, 0.22, 0.4)	(0.16, 0.35, 0.64)	(0.27, 0.45, 0.68)	(0.26, 0.4, 0.61)
6	(0, 0.1, 0.27)	(0.17, 0.36, 0.64)	(0.11, 0.22, 0.4)	(0, 0.14, 0.41)	(0, 0.11, 0.31)	(0.33, 0.5, 0.64)
7	(0.34, 0.52, 0.65)	(0, 0.14, 0.41)	(0.23, 0.36, 0.55)	(0.16, 0.35, 0.64)	(0.13, 0.28, 0.5)	(0, 0.1, 0.27)
8	(0.13, 0.26, 0.45)	(0, 0, 0.22)	(0.23, 0.36, 0.55)	(0, 0.14, 0.41)	(0.13, 0.28, 0.5)	(0.13, 0.25, 0.44)
9	(0.26, 0.41, 0.62)	(0.17, 0.36, 0.64)	(0.23, 0.36, 0.55)	(0, 0.14, 0.41)	(0.13, 0.28, 0.5)	(0.26, 0.4, 0.61)

**Table A2:** Fuzzy prioritized scores of smart technology applications.

*q*	${\tilde{s}}_{q1}$	${\tilde{s}}_{q2}$	${\tilde{s}}_{q3}$	${\tilde{s}}_{q4}$	${\tilde{s}}_{q5}$	${\tilde{s}}_{q6}$
1	(0.02, 0.08, 0.23)	(0.01, 0.06, 0.21)	(0.07, 0.2, 0.36)	(0.02, 0.06, 0.2)	(0, 0.01, 0.05)	(0, 0.01, 0.05)
2	(0.01, 0.05, 0.17)	(0, 0, 0.07)	(0.03, 0.1, 0.25)	(0.01, 0.04, 0.15)	(0.01, 0.02, 0.07)	(0, 0.01, 0.05)
3	(0.01, 0.05, 0.17)	(0.01, 0.06, 0.21)	(0.06, 0.16, 0.35)	(0.01, 0.04, 0.15)	(0, 0.01, 0.05)	(0.01, 0.02, 0.07)
4	(0.02, 0.08, 0.23)	(0.01, 0.06, 0.21)	(0.06, 0.16, 0.35)	(0.01, 0.04, 0.15)	(0.01, 0.02, 0.07)	(0, 0.01, 0.05)
5	(0, 0.02, 0.1)	(0.03, 0.1, 0.27)	(0.03, 0.1, 0.25)	(0.01, 0.04, 0.15)	(0.01, 0.02, 0.07)	(0.01, 0.02, 0.07)
6	(0, 0.02, 0.1)	(0.01, 0.06, 0.21)	(0.03, 0.1, 0.25)	(0, 0.02, 0.1)	(0, 0, 0.03)	(0.01, 0.02, 0.07)
7	(0.03, 0.1, 0.24)	(0, 0.02, 0.13)	(0.06, 0.16, 0.35)	(0.01, 0.04, 0.15)	(0, 0.01, 0.05)	(0, 0, 0.03)
8	(0.01, 0.05, 0.17)	(0, 0, 0.07)	(0.06, 0.16, 0.35)	(0, 0.02, 0.1)	(0, 0.01, 0.05)	(0, 0.01, 0.05)
9	(0.02, 0.08, 0.23)	(0.01, 0.06, 0.21)	(0.06, 0.16, 0.35)	(0, 0.02, 0.1)	(0, 0.01, 0.05)	(0.01, 0.02, 0.07)

**Table A3:** Fuzzy ideal point and fuzzy anti-ideal point.

Reference Point	${\tilde{\Lambda }}_{1}^{+/-}$	${\tilde{\Lambda }}_{2}^{+/-}$	${\tilde{\Lambda }}_{3}^{+/-}$	${\tilde{\Lambda }}_{4}^{+/-}$	${\tilde{\Lambda }}_{5}^{+/-}$	${\tilde{\Lambda }}_{6}^{+/-}$
Fuzzy ideal point	(0.03, 0.1, 0.24)	(0.03, 0.1, 0.27)	(0.07, 0.2, 0.36)	(0.02, 0.06, 0.2)	(0.01, 0.02, 0.07)	(0.01, 0.02, 0.07)
Fuzzy anti-ideal point	(0, 0.02, 0.1)	(0, 0, 0.07)	(0.03, 0.1, 0.25)	(0, 0.02, 0.1)	(0, 0, 0.03)	(0, 0, 0.03)

**Table A4:** Distances between each smart technology application and the two reference points.

*q*	${\tilde{d}}_{q}^{+}$	${\tilde{d}}_{q}^{-}$
1	(0, 0.04, 0.49)	(0, 0.14, 0.5)
2	(0, 0.15, 0.54)	(0, 0.04, 0.34)
3	(0, 0.08, 0.51)	(0, 0.09, 0.45)
4	(0, 0.06, 0.51)	(0, 0.11, 0.48)
5	(0, 0.13, 0.53)	(0, 0.1, 0.41)
6	(0, 0.14, 0.54)	(0, 0.06, 0.35)
7	(0, 0.09, 0.51)	(0, 0.11, 0.45)
8	(0, 0.13, 0.52)	(0, 0.07, 0.39)
9	(0, 0.07, 0.51)	(0, 0.11, 0.46)
